# Frequency and Predictors of Self-Reported Hypoglycemia in Insulin-Treated Diabetes

**DOI:** 10.1155/2017/7425925

**Published:** 2017-08-20

**Authors:** Samir Malkani, Anupam Kotwal

**Affiliations:** ^1^Division of Diabetes, Department of Medicine, University of Massachusetts Medical School, Worcester, MA 01655, USA; ^2^Division of Endocrinology, Diabetes, Metabolism, and Nutrition, Mayo Clinic, 200 First Street SW, Mayo 18, Rochester, MN 55905, USA

## Abstract

**Aims:**

Hypoglycemia is a limiting factor for achieving stringent glycemic control in diabetes. This study analyzes the frequency and predictors of hypoglycemia in insulin-treated diabetes in an ambulatory setting.

**Methods:**

A retrospective chart review was performed to study self-monitored blood glucose (SMBG) data for 3 months prior to a patient's HbA1c test.

**Results:**

Hypoglycemia occurred more frequently in type 1 than in type 2 diabetes; however, 19% of type 2 diabetes patients did experience at least one episode of severe hypoglycemia. For type 1 diabetes, hypoglycemia had a positive association with glycemic variability and duration of diabetes and a negative association with HbA1c and lowest blood glucose (BG). For type 2 diabetes, a positive association was noted with glycemic variability and a negative association with age and lowest BG.

**Conclusions:**

Delineating factors predisposing to hypoglycemia in type 2 diabetes is difficult. Lower HbA1c is a potential predictor of hypoglycemia in type 1 but not in type 2 diabetes. Longer duration of diabetes for type 1 and younger age for type 2 are associated with more hypoglycemia. Glycemic variability portends increased risk for hypoglycemia and should be a focus of further research.

## 1. Introduction

Hypoglycemia is a major limiting factor for achieving stringent glycemic control in diabetes. It influences self-management of diabetes with some patients accepting suboptimal glycemic control to reduce the risk of hypoglycemia [1]. Moreover, hypoglycemia is associated with significant morbidity. Severe hypoglycemia can cause seizures and arrhythmias. It is associated with dementia [2], and it has been linked with poor cardiovascular outcomes [3] and death [4]. Hypoglycemia has also been shown to trigger adrenergic discharge and the expression of proinflammatory cytokines [5].

Hypoglycemia is common in type 1 diabetes, and its occurrence increases with insulin use in type 2 diabetes [6–10]. It is a common and potentially dangerous side effect of insulin therapy; however, its occurrence is not easy to predict. The majority of our knowledge about severe hypoglycemia comes from type 1 diabetes. Data on rates and potential risk factors for severe hypoglycemia in insulin-treated type 2 diabetes are relatively scarce and conflicting. Clinical trials have reported low rates of severe hypoglycemia in type 2 diabetes [11, 12], and population-based studies have reported highly variable rates [13, 14]. The reported frequency of severe hypoglycemia in clinical trials may represent only the “tip of the iceberg” in terms of all episodes. In the case of severe hypoglycemia, most studies have used its clinical definition that was first described by the Diabetes Control and Complications Trial (DCCT), as any hypoglycemia that requires external assistance [15]. However, reporting of such episodes by patients, especially those with hypoglycemia unawareness, can be unreliable. Data derived from the frequency of treated episodes of severe hypoglycemia by emergency services are similarly inadequate to quantify the overall incidence, as only 10% of severe episodes are treated in the hospital. Data from continuous glucose monitoring (CGM) studies reveal that even asymptomatic hypoglycemia is associated with the risk of cardiac arrhythmias [16].

In patients on insulin therapy, inadequate caloric intake, exercise, renal insufficiency, and alcohol ingestion can precipitate hypoglycemia. In many episodes of hypoglycemia, a precipitating factor cannot be identified. Although some phenotypic markers are known to predispose patients to hypoglycemia, its predictors in insulin-treated diabetes have not been well studied, especially when these patients are followed on an ambulatory basis and have not been actively enrolled in a study. Hypoglycemia unawareness is common in longstanding diabetes and can lead to serious consequences in spite of lack of symptoms, and blood glucose testing or CGM can identify such episodes more reliably. To try and fill the gaps in knowledge, we conducted this study to analyze the frequency and potential predictors of self-monitored hypoglycemia in type 1 and insulin-treated type 2 diabetes in an ambulatory setting.

## 2. Material and Methods

### 2.1. Subjects with Diabetes Mellitus

We performed a cross-sectional chart review study utilizing the electronic database of the University of Massachusetts Medical School Diabetes Clinic. The clinic, which serves residents of central Massachusetts, uses an electronic diabetes management system, My Care Team™ (MCT) that captures all the patients' self-monitored blood glucose (SMBG) data. The system is able to upload readings from most commercial blood glucose monitors; data from blood glucose meters is uploaded at each clinic visit. Diabetic adults who were seen at the clinic between October 1, 2012, and September 30, 2013, were eligible if they were on insulin treatment, and their meter download on MCT showed a SMBG testing frequency of 3 times or more daily. Pregnant women were excluded. The UMass IRB approved the study proposal. Of the total sample of 3000 patients, 298 were included in the study based on the eligibility criteria. Each patient had a hemoglobin A1c (HbA1c) tested at the visit, and the SMBG data for a 3-month period prior to the clinic visit was collected. For patients who had more than one clinic visit during the study period, the most recent one was used for the purpose of data collection. Categorical variables assessed were gender, race, and presence or absence of depression, coronary artery disease (CAD), hypertension (HTN), nephropathy, and retinopathy. Nephropathy was defined as albuminuria (spot urine albumin : creatinine ratio > 300 mcg/mg) or progressive chronic kidney disease, and presence of retinopathy included all classes of detectable retinopathy. Continuous variables assessed were age, body mass index (BMI), duration of diabetes, HbA1c, daily insulin dose, number of daily injections, and SMBG data including minimum SMBG value and standard deviation (SD) around mean SMBG.

### 2.2. Identification of Hypoglycemic Episodes

Hypoglycemia was defined as a SMBG reading ≤ 70 mg/dL [17], and severe hypoglycemia as a SMBG reading ≤ 45 mg/dL. All commercial SMBG monitors provide glucose readings such that the reported result most closely matches that of a laboratory method measuring serum or plasma glucose [18]. No distinction was made between asymptomatic and symptomatic hypoglycemic episodes. We calculated the number hypoglycemic episodes for each patient from their home glucose meter upload for a 3-month period prior to their clinic visit. Sequential SMBG readings meeting hypoglycemia criteria recorded within a 2-hour period were considered as a single episode.

### 2.3. Statistical Analysis

A database was created with data for all the variables, and statistical analysis was performed using the software SPSS. As the independent variables were not distributed normally, nonparametric tests of significance were utilized. Mann–Whitney *U* test was used to assess the association of categorical independent variables with dependent variables (frequency of hypoglycemia and severe hypoglycemia). For continuous variables, Spearman's correlation and Wilcoxon signed-rank test were used for univariate analysis. *p* value ≤ 0.05 was considered significant. All probabilities were two tailed. Multivariate linear regression (as dependent variables were continuous) was then used for all the associations that were significant on univariate analysis to remove the effect of confounders. Two separate models were created using the frequencies of all hypoglycemic episodes and severe hypoglycemic episodes as dependent variables.

## 3. Results

### 3.1. Study Sample

A total of 298 patients with diabetes on insulin were included for analysis. This included 133 patients with type 1 and 165 patients with type 2 diabetes. Of the 133 subjects with type 1 diabetes, 47 (35.3%) were using an insulin pump and the rest were on multiple daily injections (MDI). The characteristics of the subjects including mean age, gender distribution, and diabetes duration are shown in Table 1. The group with type 1 diabetes had a significantly lower age, BMI, and duration of diabetes as well as a significantly lower percentage of individuals suffering from hypertension, CAD, and nephropathy as compared to the group with type 2 diabetes. This group had a significantly higher mean HbA1c, number of daily insulin injections, daily insulin dose, and SD around mean SMBG (indicator of glycemic variability) as compared to the group with type 2 diabetes. There was no statistically significant difference in regard to gender, daily insulin dose adjusted for BMI, and the percentage of individuals with depression or retinopathy between the two groups. The characteristics of the individuals with insulin-treated diabetes that were excluded from the study were similar to those that were included.

### 3.2. Frequency of Hypoglycemia

All patients with type 1 diabetes and 82% of patients with type 2 diabetes experienced at least one episode of hypoglycemia. The corresponding numbers for severe hypoglycemia were 68% for type 1 and 19% for type 2 diabetes. 88% of those with type 1 experienced more than 10 hypoglycemic episodes compared to 30% with type 2 diabetes. Those with type 1 diabetes recorded a total of 4583 hypoglycemic episodes that corresponds to a rate of 137.8 (95% CI 135.9 and 139.8) episodes per person per year. 643 (14%) of these were severe, corresponding to 19.3 (95% CI 18.6 and 20.1) severe episodes per person per year. In those with type 2 diabetes, a total of 1517 hypoglycemic episodes were recorded; of which, 119 (8%) were severe. This corresponds to an event rate per person per year for hypoglycemia and severe hypoglycemia of 36.8 (95% CI 35.9, 37.7) and 2.9 (95% CI 2.6, 3.2), respectively. Less than 1% of individuals with type 2 diabetes reported greater than 10 severe hypoglycemic episodes compared to almost 14% with type 1 diabetes. The frequencies of both all and severe hypoglycemic episodes were significantly higher for type 1 as compared to type 2 diabetes (*p* = 0.000 for both).

### 3.3. Predictors of Hypoglycemia in Type 1 Diabetes

On univariate analysis initially by correlation (for continuous variables) and then tests of significance (Tables 2 and 3), the frequencies of all and severe hypoglycemia had a significant negative association with the mean HbA1c (*p* = 0.000 for both), the lowest BG reading recorded (*p* = 0.000 for both), BMI (*p* = 0.000 for both), and age (*p* = 0.000 for both). The frequencies of all and severe hypoglycemia demonstrated a significant positive association with the duration of diabetes (*p* = 0.000 for both), daily insulin dose (*p* = 0.003 and 0.000, resp.), and glycemic variability (*p* = 0.000 for both). There was a positive association with the number of daily insulin injections that was statistically significant only for all hypoglycemia (*p* = 0.000).

Multivariate linear regression analysis (Table 4) which adjusted for independently associated variables revealed a significant positive association of the frequencies of all and severe hypoglycemia with glycemic variability and duration of diabetes and a significant negative association with HbA1c. When the lowest BG reading was included in this analysis, it continued to be significantly negatively associated with both all and severe hypoglycemia. The association with other variables like age, BMI, number of daily insulin injections, and daily insulin dose did not achieve significance in the regression analysis.

### 3.4. Predictors of Hypoglycemia in Insulin-Treated Type 2 Diabetes

On univariate analysis, the frequencies of all and severe hypoglycemia had a significant negative association with the lowest BG reading recorded (*p* = 0.000 for both), BMI (*p* = 0.000 for both), and age (*p* = 0.000 for both). There was a negative association with HbA1c that was statistically significant only for severe hypoglycemia (*p* = 0.000). The frequencies of all and severe hypoglycemia had a significant positive association with the duration of diabetes (*p* = 0.000 for both), daily insulin dose (*p* = 0.000 for both), number of daily insulin injections (*p* = 0.000 for both), and glycemic variability (*p* = 0.000 for both). The frequency of all hypoglycemia had a significant positive association with the presence of depression (*p* = 0.047).

Multivariate linear regression analysis (Table 4), which adjusted for independently associated variables, revealed a significant negative association of the frequency of all hypoglycemia with age. The association with other variables seen on univariate analysis did not achieve significance during regression analysis. However, when the lowest BG reading was included in this analysis, it continued to be significantly negatively associated with both all and severe hypoglycemia, and the positive association of all hypoglycemia with glycemic variability achieved statistical significance.

When comparing individuals with type 1 diabetes using an insulin pump versus those on MDI, there was no statistically significant difference between the frequencies of all or severe hypoglycemia and mean HbA1C value. In regard to different therapeutic regimens, there was no statistically significant difference in hypoglycemia frequency when comparing different MDI regimens for either type 1 or type 2 diabetes.

## 4. Discussion

Although hypoglycemia is a common complication of insulin treatment in diabetes, very few studies have evaluated the frequency and predictors of self-reported hypoglycemia on an ambulatory basis, especially in insulin-treated type 2 diabetes. The present study examined self-reported hypoglycemia in a sample of patients with insulin-treated diabetes who were being managed at a university hospital diabetes clinic and accurately represented the population base. Our results are based on hypoglycemia recorded by the subject whether or not they experienced symptoms. SMBG frequency of at least 3 times a day reduced the likelihood of missing any episodes of hypoglycemia. The over recording of hypoglycemia in the initial data was corrected by counting SMBG readings ≤ 70 mg/dL recorded within a 2-hour period as one hypoglycemic episode unless there was a reading > 70 mg/dL in between them. It is not surprising that our patients with type 1 diabetes reported more hypoglycemic episodes as compared to those with type 2 diabetes. However, compared with previous studies of both type 1 [19, 20] and type 2 diabetes [11, 14, 21], the event rates and the proportion of subjects experiencing hypoglycemic events in our study were higher, especially in type 2 diabetes with 19% experiencing an episode of severe hypoglycemia. The possible reasons for this could be the longer duration of diabetes in our patients, being followed on an ambulatory basis as compared to being enrolled in a clinical trial, and the fact that many other studies have defined severe hypoglycemia on symptoms and not BG values. The rates of severe hypoglycemia in insulin-treated type 2 diabetes are reported to be low [11, 14, 21], but these have been recorded in the context of clinical trials and often in people with a short duration of insulin therapy.

Insulin use has been linked to higher likelihood of experiencing hypoglycemia; however, within the group of insulin-treated diabetes, the predictors of hypoglycemia remain unclear. In type 1 diabetes, a negative association of hypoglycemia with HbA1c has been reported by several studies [15, 22, 23]. In type 2 diabetes, either no association or a positive association of hypoglycemia with HbA1c has been reported [8, 20, 24] except in the PREDICTIVE trial which demonstrated a negative association between the two in both type 1 and type 2 diabetes [23]. Within the intensive treatment arm of the ACCORD trial for type 2 diabetes, a higher HbA1c and lower change in HbA1c from baseline were associated with a higher rate of occurrence of hypoglycemia [25]. In our study, after adjusting for independent variables, lower HbA1c was a potential predictor of hypoglycemia in type 1 but not in type 2 diabetes. Glycemic variability has been assessed by studies using different parameters, SD around mean BG being the best demonstrated for SMBG data. In our study, this was a significant predictor for hypoglycemia. A history of previous severe hypoglycemia has been reported to be a risk factor for severe hypoglycemia [15, 26]. Similarly, our study found a significant association between experiencing a low PG reading and increased frequency of all and severe hypoglycemic episodes in both type 1 and type 2 diabetes. Also, the distribution of hypoglycemia in our study was highly skewed with a small proportion of patients accounting for the majority of episodes.

In the UKPDS trial, hypoglycemia was more frequent in younger patients, females, non-obese patients and those with positive islet cell antibodies [11]. Our study also showed a higher frequency of hypoglycemia in younger type 2 diabetes patients, but there was no significant association with gender or BMI in regression analysis. However these two studies are not exactly comparable as we studied only insulin-treated diabetics with longer diabetes duration. Other studies have reported hypoglycemia in type 2 diabetes to be linked to longer duration of diabetes [7, 20] which we found on univariate but not multivariate analysis. The increased occurrence of hypoglycemia in patients with longer duration of diabetes could be related to the accompanying hypoglycemia-associated autonomic failure (HAAF). Depression has been associated with severe hypoglycemia in type 2 diabetes [27]; in our study, this association was observed on univariate analysis but did not hold up in multivariate regression analysis. In type 2 diabetes, a lower BMI is associated with hypoglycemia in some studies [23], and in our cohort, this relationship was observed only in univariate, but not in multivariate analysis. Our observation of higher rates of hypoglycemia in younger type 2 diabetes patients can be explained by the higher insulin sensitivity in this group. There was no significant difference in the frequency of hypoglycemia or mean HbA1c between those with type 1 diabetes that were on an insulin pump versus those on MDI; however the study was not powered to assess this. Similar findings have been reported by a recent meta-analysis [28].

## 5. Conclusions

Insulin treatment of diabetes increases the likelihood of experiencing hypoglycemia. We studied a cohort of patients with relatively well-controlled diabetes in terms of their HbA1c values, who had diabetes for a long duration thus increasing their risk of HAAF. Even though hypoglycemia is more common in type 1 diabetes, it continues to remain an important complication of insulin treatment in type 2 diabetes. In type 1 diabetes, striving for a lower HbA1c increases the risk of hypoglycemia. In addition, increased glycemic variability and longer duration of diabetes are significant potential predictors of hypoglycemia. In type 2 diabetes, factors predisposing to hypoglycemia are interdependent and we found it difficult to delineate them separately. However, we did find higher glycemic variability to be associated with a higher frequency of hypoglycemic episodes in this group. There seems to be a positive association of hypoglycemia with depression in type 2 diabetes, which needs to be further explored. In our study, younger insulin-treated type 2 diabetics experienced more hypoglycemic episodes, which is contrary to some other studies. Experiencing a hypoglycemic episode increases the risk of more hypoglycemia in both type 1 and type 2 diabetes. Very frequent hypoglycemia occurs in a small proportion of patients, and these are the ones that require the greatest attention to avoid hypoglycemia. Glycemic variability, which is an indicator of poor glycemic control rather than tight glycemic control, portends increased risk for hypoglycemia. Studies that focus on glycemic variability and interventions to prevent hypoglycemia in high-risk patients are needed.

## Figures and Tables

**Table 1:** Characteristics of patients with insulin-treated diabetes participating in the study.

Patient characteristics	All patients Mean ± SD, range or *n* (%) patients	Type 1 diabetes Mean ± SD, range or *n* (%) patients	Type 2 diabetes Mean ± SD, range or *n* (%) patients	*p* value
Number of patients (%)	298 (100)	133 (44.6)	165 (55.4)	—
Age (years)	60.54 ± 16.64	52.11 ± 17.19	67.33 ± 12.63	0.001
Males	153/145	62 (46.6)	83 (50)	0.606
BMI (kg/m^2^) [mean ± SD]	30.38 ± 7.45	26.38 ± 4.66	33.58 ± 7.73	0.001
Known duration of diabetes (years) [mean ± SD]	21.43 ± 14.62	25.26 ± 16.75	18.11 ± 11.58	0.001
Daily insulin dose (units/day) [mean ± SD]	66.48 ± 56.05	49.04 ± 26.91	74.59 ± 63.80	0.001
Daily insulin dose/BMI (units/kg/m^2^/day) [mean ± SD]	2.06 ± 1.35 (*n* = 210)	1.84 ± 0.91 (*n* = 66)	2.16 ± 1.52 (*n* = 144)	0.307
HbA1c (%) [mean ± SD]	7.77 ± 1.25	7.94 ± 1.94	7.64 ± 1.27	0.032
<7		31 (23.3)	48 (28.9)	
7-8		36 (27.1)	59 (35.5)	
>8		66 (49.6)	58 (34.9)	
SD around mean SMBG	68.71 ± 17.04	78.93 ± 2.04	60.47 ± 1.69	0.001
Lowest SMBG reading [mean ± SD]	49.69 ± 15.78	40.98 ± 9.54	56.71 ± 16.33	0.001
Number of daily insulin injections [mean ± SD]	3.87 ± 0.93 (median 4, range 1–6)	4.12 ± 0.48 (median 4, range 2–6)	3.73 ± 1.08 (median 4, range 1–5)	0.001
MDI		86 (64.7)	165 (100)	—
Insulin pump		47 (35.3)	None	
HTN	170 (57.0)	47 (35.3)	123 (74.5)	0.001
CAD	74 (24.8)	19 (14.3)	55 (33.3)	0.001
Nephropathy	102 (34.2)	27 (20.3)	75 (45.2)	0.001
Retinopathy (*n*, %)	63 (21.1)	32 (24.1)	31 (18.8)	0.334
Depression (*n*, %)	49 (16.4)	16 (12.0)	33 (20.0)	0.091
Caucasians (*n*, %)	265 (88.9)	128 (96.2)	137 (83.1)	0.001
All hypoglycemic episodes	6100 events	4583 events in 133 (100%) patients	1517 events in 136 (82%) patients	0.000
All hypoglycemic episodes per person per year [OR (95% CI)]	81.9 (80.0 to 82.9)	137.8 (135.9 to 139.8)	36.8 (35.9 to 37.7)	0.000
No hypoglycemic episodes	29 (9.7%)	0 (0%)	29 (17.6%)	—
1–10 hypoglycemic episodes in 3 months	269 (90.3%)	16 (12%)	86 (52.1%)	—
>10 hypoglycemic episodes in 3 months	167 (56.0%)	117 (88%)	50 (30.3%)	—
Severe hypoglycemic episodes	762	643 events in 91 (68%) patients	119 events in 32 (19%) patients	0.000
Severe hypoglycemic episodes per person per year [OR (95% CI)]	10.2 (9.9 to 10.6)	19.3 (18.6 to 20.1)	2.9 (2.6 to 3.2)	0.000
No severe hypoglycemic episodes	175 (58.7%)	42 (31.6%)	133 (80.6%)	—
1–10 severe hypoglycemic episodes in 3 months	123 (41.3%)	73 (54.9%)	31 (18.8%)	—
>10 severe hypoglycemic episodes in 3 months	19 (6.4%)	18 (13.5%)	1 (0.6%)	—

**Table 2:** Results of nonparametric test (Mann–Whitney *U* test) for association of categorical independent variables with frequencies of hypoglycemia and severe hypoglycemia.

Categorical variables	Type 1 diabetes				Type 2 diabetes			
Mann–Whitney *U* test	Hypoglycemia [mean ± SD]	*p* value	Severe hypoglycemia [mean ± SD]	*p* value	Hypoglycemia [mean ± SD]	*p* value	Severe hypoglycemia [mean ± SD]	*p* value
Females	37.96 ± 26.40	0.188	5.69 ± 7.99	0.523	8.16 ± 9.96	0.617	0.88 ± 2.66	0.412
Males	30.45 ± 19.31		3.85 ± 5.22		10.22 ± 11.91		0.57 ± 1.72	
Nephropathy absent	36.45 ± 24.62	0.089	5.10 ± 7.08	0.162	9.57 ± 11.84	0.581	0.91 ± 2.69	0.487
Nephropathy present	26.63 ± 17.16		3.78 ± 6.02		8.75 ± 9.95		0.49 ± 1.50	
Retinopathy absent	35.85 ± 24.14	0.258	4.94 ± 7.03	0.524	9.22 ± 11.68	0.109	0.81 ± 2.43	0.551
Retinopathy present	30.06 ± 21.49		4.50 ± 6.46		9.06 ± 7.53		0.35 ± 1.02	
On MDI	32.92 ± 23.43	0.224	4.85 ± 7.24	0.543	9.30 ± 11.02	0.085	0.73 ± 2.25	—
On insulin pump	37.28 ± 23.84		4.81 ± 6.23		0.50 ± 0.71		0.00	
HTN absent	35.23 ± 24.09	0.648	5.08 ± 6.94	0.377	10.48 ± 11.60	0.324	0.74 ± 1.81	0.647
HTN present	33.04 ± 22.79		4.38 ± 6.80		8.76 ± 10.80		0.71 ± 2.37	
CAD absent	35.69 ± 23.23	0.063	5.12 ± 7.09	0.132	9.50 ± 11.08	0.367	0.76 ± 2.42	0.783
CAD present	27.05 ± 24.92		3.10 ± 5.22		8.58 ± 10.91		0.64 ± 1.83	
Other race	24.60 ± 11.33	0.435	1.40 ± 1.34	0.284	10.11 ± 12.25	0.913	1.28 ± 2.92	0.326
Caucasians	34.84 ± 23.87		4.97 ± 6.97		9.01 ± 10.76		0.60 ± 2.06	
Depression absent	34.63 ± 24.07	0.912	4.69 ± 6.86	0.484	8.56 ± 10.72	0.047	0.67 ± 2.29	0.366
Depression present	33.19 ± 20.21		5.87 ± 7.13		11.73 ± 11.89		0.94 ± 2.03	
Different categories of A1c (<7, 7-8, >8)		0.001		0.052		0.507		0.363

**Table 3:** Results of nonparametric tests Spearman's correlation (to calculate correlation coefficient) and Wilcoxon signed-rank test (to assess significance with *p* value) for association of continuous independent variables with frequencies of hypoglycemia and severe hypoglycemia.

Continuous variables	Type 1 diabetes				Type 2 diabetes			
	Hypoglycemia		Severe hypoglycemia		Hypoglycemia		Severe hypoglycemia	
	Correlation coefficient *ρ*	*p* value	Correlation coefficient *ρ*	*p* value	Correlation coefficient *ρ*	*p* value	Correlation coefficient *ρ*	*p* value
Age (years)	−0.2	0.000	−0.2	0.000	−0.03	0.000	−0.01	0.000
BMI	−0.1	0.005	−0.1	0.000	−0.1	0.000	−0.2	0.000
Duration of diabetes	+0.2	0.000	+0.2	0.000	+0.2	0.000	+0.2	0.000
Daily insulin dose	0.1 (*n* = 66)	0.003	0.1 (*n* = 46)	0.000	0.1 (*n* = 119)	0.000	0.01 (*n* = 29)	0.000
Daily insulin injections	+0.2	0.000	+0.2	0.14	+0.04	0.000	+0.1	0.000
Lowest BG reading	−0.7	0.006	−0.9	0.000	−0.8	0.000	−0.7	0.000
SD around mean BG	+0.1	0.000	+0.2	0.000	+0.2	0.000	+0.2	0.000
HbA1c	−0.4	0.000	−0.2	0.000	−0.1	0.456	−0.04	0.000

**Table 4:** Multivariate regression analysis using hypoglycemic episodes and severe hypoglycemic episodes as dependent variables and variables that achieved significance on univariate analysis as independent variables.

Variables significant on univariate analysis	Type 1 diabetes		Type 2 diabetes	
	Hypoglycemia (beta; *p* value)	Severe hypoglycemia (beta; *p* value)	Hypoglycemia (beta; *p* value)	Severe hypoglycemia (beta; *p* value)
Age	−0.25; 0.28	−0.09; 0.23	−0.26; 0.032	−0.01; 0.84
BMI	1.27; 0.14	0.11; 0.71	−0.27; 0.10	−0.03; 0.41
Duration of diabetes	0.46; 0.042	0.16; 0.037	0.18; 0.11	−0.01; 0.65
Total insulin dose	0.24; 0.11	0.02; 0.72	0.01; 0.74	0.002; 0.79
Number of daily injections	1.95; 0.76	—	−0.89; 0.44	0.05; 0.84
SD around mean BG	0.43; 0.022	0.15; 0.023	0.07; 0.22	0.01; 0.50
HbA1c	−9.69; 0.010	−3.19; 0.015	—	−0.09; 0.71
Depression	—	—	−1.63; 0.61	—
